# Novel EAAT2 activators improve motor and cognitive impairment in a transgenic model of Huntington’s disease

**DOI:** 10.3389/fnbeh.2023.1176777

**Published:** 2023-06-07

**Authors:** Akanksha Bhatnagar, Visha Parmar, Nicholas Barbieri, Frank Bearoff, Felice Elefant, Sandhya Kortagere

**Affiliations:** ^1^Department of Biology, Papadakis Integrated Sciences Building, Drexel University, Philadelphia, PA, United States; ^2^Department of Microbiology and Immunology, Drexel University College of Medicine, Philadelphia, PA, United States

**Keywords:** EAAT2 activators, GTS467, Huntington’s disease, learning and memory, motor impairment, survival

## Abstract

**Introduction:**

Glutamate excitotoxicity is causal in striatal neurodegeneration underlying motor dysfunction and cognitive deficits in Huntington’s disease (HD). Excitatory amino acid transporter 2 (EAAT2), the predominant glutamate transporter accounting for >90% of glutamate transport, plays a key role in preventing excitotoxicity by clearing excess glutamate from the intrasynaptic cleft. Accordingly, EAAT2 has emerged as a promising therapeutic target for prevention of neuronal excitotoxicity underlying HD and other neurodegenerative diseases.

**Methods:**

We have previously designed novel EAAT2 positive allosteric modulator GT951, GTS467, and GTS551, with low nanomolar efficacy in glutamate uptake and favorable pharmacokinetic properties. In this study, we test the neuroprotective abilities of these novel EAAT2 activators *in vivo* using the robust *Drosophila* HD transgenic model expressing human huntingtin gene with expanded repeats (Htt128Q).

**Results:**

All three compounds significantly restored motor function impaired under HD pathology over a wide dose range. Additionally, treatment with all three compounds significantly improved HD-associated olfactory associative learning and short-term memory defects, while GT951 and GTS551 also improved middle-term memory in low-performing group. Similarly, treatment with GT951 and GTS551 partially protected against early mortality observed in our HD model. Further, treatment with all three EAAT2 activators induced epigenetic expression of EAAT2 *Drosophila* homolog and several cognition-associated genes.

**Conclusion:**

Together, these results highlight the efficacy of GT951, GTS467 and GTS551 in treating motor and cognitive impairments under HD pathology and support their development for treatment of HD.

## Introduction

Huntington’s disease (HD) is a chronic inherited neurodegenerative disease caused by CAG trinucleotide repeat expansion in the huntingtin (*htt*) gene on chromosome 4 (Bates et al., 2015; Jimenez-Sanchez et al., 2017). These repeats are translated into an abnormally long polyglutamine tract, resulting in progressive loss of medium spiny neurons (MSN) within the striatum as well as cortical neurons that project to the striatum (Snowden, 2017; Zheng and Kozloski, 2017). HD patients develop heterogenous clinical symptoms characterized by motor, cognitive and psychiatric impairments (Novak and Tabrizi, 2010; Ghosh and Tabrizi, 2018). The age at onset of HD is typically inversely correlated to CAG repeat length with an average onset age around 30–50 years, however, rare cases of juvenile HD have been reported (Langbehn et al., 2010; Novak and Tabrizi, 2010; Ghosh and Tabrizi, 2018). In United States alone, presently 40,000 people are estimated to be living with HD and another 200,000 are at risk of developing HD (Perkins, 2017; Yohrling et al., 2020), therefore imposing a serious public health burden. Although there has been significant advances in the understanding of HD pathogenesis (Dash and Mestre, 2020), there are currently no disease modifying treatments for HD and only symptomatic treatments remain (Pan and Feigin, 2021). These symptomatic treatments do not work optimally for all disease states and have significant side effects such as active depression, suicidal ideations and psychosis (Frank and Jankovic, 2010; Coppen and Roos, 2017; Gibson and Claassen, 2021).

Several compelling studies have implicated the causal role of glutamate excitotoxicity in striatal neurodegeneration associated with HD pathogenesis (Beal, 1992; Massieu and Garcia, 1998; Fan and Raymond, 2007; Sánchez et al., 2008; Jamwal and Kumar, 2019; Fontana et al., 2023). The excitotoxicity hypothesis of HD originated from an earlier observation that glutamate receptor agonists induce neuronal loss and reproduce HD-associated symptoms (Beal et al., 1986) while glutamate receptor antagonists protected against excitotoxicity in a rodent model of HD (Greene et al., 1993). Additionally, HD transgenic mouse models have since revealed increased postsynaptic glutamatergic NMDA receptor activity in neurons (Cepeda et al., 2001; Zeron et al., 2002) and decreased expression of glutamate transporters (Arzberger et al., 1997), suggesting glutamatergic neurotransmission is greatly dysregulated in HD. The HD excitatory hypothesis also supports the selective loss of MSNs in the striatum that are particularly susceptible to excitotoxic neuronal death in HD (Zeron et al., 2002). Glutamate excitotoxicity results from prolonged receptor exposure or excessive glutamate concentrations in the intrasynaptic cleft that can be prevented by efficient glutamate clearance (Parsons et al., 2016). In mammalian brains, a family of five distinct sodium-dependent glutamate transporters have been identified and termed Excitatory Amino Acid Transporter (EAAT)1–5 (O’Donovan et al., 2017). The predominant glutamate transporter, EAAT2, is abundantly expressed in the brain representing 1% of total brain protein (Danbolt, 2001) and accounts for a more than 90% of glutamate transport, although there is evidence of EAAT1 present on axonal terminals in hippocampus may contribute to glutamate uptake specially when devoid of adjacent astrocyte processes (Foran and Trotti, 2009; Petr et al., 2015). Accordingly, EAAT2 is essential for maintaining low extracellular glutamate levels and its dysfunction has been linked with excitotoxicity and neurodegeneration (Rothstein et al., 1996; Arzberger et al., 1997; Kim et al., 2011). In mice, EAAT2 knockdown has been shown to elevate glutamate concentrations triggering seizures, neuronal death, and early mortality (Tanaka et al., 1997). Additionally, evidence from several HD transgenic models demonstrates mutant htt expression selectively downregulates EAAT2 mRNA and protein levels that ultimately results in extracellular glutamate accumulation, excitotoxicity, and neuronal cell death (Behrens et al., 2002; Liévens et al., 2005; Shin et al., 2005; Bradford et al., 2009; Meunier et al., 2016). Finally, activating EAAT2 expression has been shown to ameliorate neurodegenerative phenotypes in HD mice models, highlighting the neuroprotective capabilities of EAAT2 activation (Miller et al., 2008; Estrada-Sánchez et al., 2009). Consequently, EAAT2 has emerged as a novel promising therapeutic target for prevention of neuronal excitotoxicity underlying HD and other neurodegenerative diseases (Kim et al., 2011; Limpert and Cosford, 2014; Takahashi et al., 2015).

Excitatory amino acid transporter 2 expression and function can be pharmacologically modulated by regulating its transcription, translation, or glutamate transport efficiency. Since NF-kB and CREB have been elucidated as intrinsic activators for transcriptional regulation of EAAT2, several drugs including tamoxifen (Karki et al., 2013), raloxifene (Karki et al., 2014b), and ceftriaxone (Lee et al., 2008) have shown to induce EAAT2 expression via NF-kB and CREB activation. Similarly, different classes of histone deacetylase inhibitors (Yoshida et al., 1990; Karki et al., 2014a; Lapucci et al., 2017) have been shown to epigenetically induce EAAT2 transcription and promotor activity. Additionally, small molecule pyridazine derivatives, such as LDN-212320 (Kong et al., 2014), upregulate EAAT2 post-transcriptionally by enhancing EAAT2 translation. Although transcriptional and translational EAAT2 activators are promising in preventing excitotoxicity and neurodegeneration, low efficacy and toxicity profiles of these compounds have limited their utility in the clinic (Lin et al., 2012). Alternatively, pharmacologically stimulating glutamate uptake by EAAT2 provides a safe yet rapid approach to prevent acute excitotoxicity. Crystal structures of a bacterial glutamate transporter homologue, GltPh, have since revealed specific structural elements that are critical for transport stimulation (Yernool et al., 2004; Reyes et al., 2009). In this regard, we have previously utilized the structural knowledge from bacterial GltPh in a hybrid structure-based approach (Kortagere and Welsh, 2006) to develop a range of novel EAAT2 positive allosteric modulators (Kortagere et al., 2017). A novel series of EAAT2 activator compounds exemplified by GT951 were identified in the screen for their potency and selectivity to EAAT2 for enhancing glutamate uptake (EC_50_ = 0.8 ± 0.3 nM) (Kortagere et al., 2017). GT951 stimulates glutamate transport by interacting with residues forming the interface between the trimerization and transport domains. Additionally, GT951 enhanced glutamate translocation rate in cultured astrocytes without affecting substrate interaction, suggesting it may be acting at an allosteric site to the substrate binding pocket of EAAT2. Since GT951 mediated glutamate transport enhancement provides a desirable approach for acutely preventing glutamate excitotoxicity and inhibiting neuronal cell death, we further optimized the pharmacokinetics properties of GT951 and synthesized novel molecules with altered structural core (Das et al., 2022). The EAAT2 activators–GT951, GTS467 and GTS511, have low nanomolar efficacy in the glutamate uptake assay, improved half-life and higher bioavailability in plasma and the brain under all three routes of administration in rats (Das et al., 2022). Next, to test these activators *in vivo*, we utilized *Drosophila melanogaster* that has been extensively used to study HD pathology and disease mechanisms. Expression of expanded human Htt polQ peptides in *Drosophila* has previously been shown to result in a significant decrease in mRNA and protein levels of dEAAT1, the *Drosophila* homolog of mammalian EAAT2, suggesting at least a partial epigenetic repression of dEAAT1 expression in the *Drosophila* HD model (Liévens et al., 2005). Additionally, *dEAAT1* inactivation results in several neurological phenotypes, including hyperexcitability, oxidative stress, decreased life span and neuropil degeneration (Rival et al., 2004). Interestingly, *dEAAT1* loss induced hyperexcitability is partially rescued by insertion of human EAAT2, suggesting the hyperexcitability behavior is at least in part a consequence of glutamate transport deficiency and EAAT-associated glutamate uptake functions are conserved between *Drosophila* and mammals (Rival et al., 2004). Furthermore, *Drosophila* has been widely used as a model organism to test modulation of glutamate-mediated neurodegeneration (Agrawal et al., 2005; Martin and Krantz, 2014; Chakravorty et al., 2022). For example, riluzole, an anti-excitotoxic agent that increases glutamate uptake has been shown to rescue memory deficits in *Drosophila* (Matsuno et al., 2019). Here, we test the neuroprotective abilities of these compounds in improving HD-associated locomotion, learning/memory impairment, and longevity phenotypes *in vivo* using a *Drosophila* HD transgenic model. We also tested if GT951 regulates expression of EAAT2 and other genes critical for cognition.

## Materials and methods

### Fly strains and crosses

All fly lines were raised on standard yeast *Drosophila* medium at 25°C with 12/12-h light/dark cycle. UAS-Gal4 targeted gene expression system was used to model HD in *Drosophila* as previously described (Romero et al., 2008; Beaver et al., 2020). The pan-neuronal driver fly line (elav^*C*155^) was used to target expression of human full-length huntingtin gene with 128 glutamine repeats [Htt(128Q)] in all neurons. Model validation was performed by confirming expression of Htt(128Q) transgene in the progeny using real-time PCR. Elav alone was used as a control. All fly lines were obtained from Bloomington *Drosophila* Stock Center (Bloomington, IN).

### Drug delivery

The drugs were incorporated into the fly food and fed to the progeny larvae and flies. DMSO was used as a vehicle to prepare drug solutions. The fly food was prepared under standard conditions and aliquoted into individual vials. The drug solutions were then added to the fly vials with final DMSO concentration ≤0.01%. For control, DMSO alone was added. The parent crosses were set in the drug food vials so that the resulting progeny can continue feeding on the drug since the early developmental stage.

### Larval line crossing assay

Larval locomotion function was analyzed as previously described (Nichols et al., 2012; Beaver et al., 2020). The line crossing apparatus consisted of a petri dish containing 2.5% agar positioned on a 0.5 cm^2^ grid paper. Wandering third instar of either sex larvae were collected from fly vials and rinsed with distilled water. The larvae were placed in the center of petri dish and allowed to acclimate for 3 min. After initial acclimation, the number of grid lines passed by the head of the larva in 30 s were recorded. Data from at least 30 larvae were collected. For locomotion assay, the drugs were tested at five drug concentrations: two-fold below EC50, one-fold below EC50, at EC50, one-fold above EC50, and two-fold above EC50. The most effective concentration was determined for each drug that was tested in the following behavioral assays.

### Olfactory learning and memory assay

Before proceeding with the testing, larval sensory reflexes were evaluated using olfactory, gustatory, and speed assays. Third instar larvae of either sex reared on food with either drugs or vehicle were washed and acclimatized on agar plates. For olfactory assay, larvae were placed in the center of freshly prepared 2.5% agar plates with linalool (LIN) odor (2.5 μL) on one side and none on the other side, and olfactory response was recorded for 3 min (*n* = 30). For gustatory assay, the larvae were placed in the center of a partitioned petri dish with 0.5% sucrose (SUC) in one half and 0.5% distilled water (DW) on the other half, and the gustatory response was assessed for 3 min (*n* = 30). For speed test, larvae were placed horizontally along the center of freshly prepared 2.5% agar plates with coordinate grid layout taped to the bottom. Larval speed (mm/s) was recorded for 1 min using the Tracker video analysis and modeling tool (Open Source Physics) (*n* = 30).

Larval cognitive performance is assessed via an improved protocol for a single odor paradigm for olfactory associative learning and memory retention (Honjo and Furukubo-Tokunaga, 2005). Larvae were first conditioned to associate the given undiluted odor, linalool (LIN, Nacalai, Tokyo, Japan), to an appetitive gustatory reinforcer, sucrose (SUC). Freshly prepared 2.5% agar plates with either 1 M SUC or distilled water (control) were spread over the agar and 10 μl LIN was placed on the lid of the petri dish. At least 50 third instar larvae per run were transferred to the olfactory training plate for 30 min. After the training period, the larvae were tested for olfactory learning response (0-min mark) on the test plate by placing contained LIN odor (2.5 μl) on one side and none on the other side. Based on their chosen side and speed, larvae were classified as high performers (odor side, <1 min), low performers (odor side, 1–3 min), or non-performers (non-odor side, 1–3 min). The larvae were collected and placed into separate groups for the rest of the assay. Each group was placed in a resting plate for 30 min and separately tested for their olfactory memory retention at 30, 60, and 90 min. For each run, the responsive index (RI) was calculated as (number of larvae in the odor area–number of larvae in the control area)/total number for larvae counted. For each group, ΔRI was calculated as the difference in RI of LIN/SUC and control LIN/DW. The locomotion speed values calculated from the speed test were used to normalize ΔRIs for all vehicle and drug treatments.

### Longevity assay

The lifespan of adult flies was determined using the *Drosophila* longevity assay (Linford et al., 2013). The parent crosses were set in fly bottles with food containing either drug solutions drug solution or DMSO vehicle (control). For each drug, the most effective drug concentration determined via locomotion assay was used. Newly enclosed progeny adult flies of either sex were collected within a 24-h period and transferred to food vial containing respective drug or DMSO solutions. A total of 20 flies were placed per food vial with an equal male: female ratio with a total of 160 flies tested per genotype. This day was marked as “Day 0.” The flies were then transferred to new vials containing fresh food with respective drug or DMSO solutions every 2 days and the surviving flies were counted. The experiment was continued until there were no surviving flies.

### qRT-PCR

Third instar larvae were reared on food with either the most effective drug concentration determined via locomotion assay or vehicle. 30 larval heads per sample were preserved in RNAlater (Thermo Fisher) and processed into RNA using TRIzol Reagent (Thermo Fisher). DNA was then digested with the TURBO DNA-free Kit (Thermo Fisher) and RNA was converted to cDNA with the High-Capacity cDNA Reverse Transcription Kit (Applied Biosystems). Gene expression was assessed with a QuantStudio 6 instrument (Thermo Fisher). Primer sets were designed by NCBI/Primer-BLAST (Supplementary Table 1). Each biological sample was analyzed in triplicate using *RpL32* as a housekeeping gene (ΔCT) and the relative values were normalized to the Vehicle treatment group using the 2^–ΔΔ^ ^CT^ method (Livak and Schmittgen, 2001).

### Statistical analysis

Data was analyzed using GraphPad Prism 9.4.0 (GraphPad Software, San Diego, CA, USA). The individual statistical test used, sample size, and results values are indicated in each figure legend. Significance levels reported are indicated as *p < 0.05; **p < 0.01, ***p < 0.001, and ****p < 0.0001.

## Results

### GT951, GTS467, and GTS551 bind to dEAAT1 at a homologous allosteric site to EAAT2

The three-dimensional structure of the *Drosophila* dEAAT1 has not been resolved to date. To evaluate if our mammalian EAAT2 activators GT951, GTS467, and GTS551 bind to dEAAT1, the homology between dEAAT1 and EAAT2 was determined. Protein sequence alignment of dEAAT1 and EAAT2 showed 54% sequence similarity and 36% identity suggesting significantly high homology between two proteins. A three-dimensional structural model of dEAAT1 was predicted using alpha fold (Jumper et al., 2021) and the model was refined using energy minimization and molecular dynamics simulation with amber force field and amber charges adopted in the modeling program molecular operating environment (MOE 2022.02). The resulting structural model of dEAAT1 was used for docking GT951, GTS467, and GTS551 using GOLD program (Jones et al., 1997). Results from docking studies showed similar or slightly better docking scores for these molecules at dEAAT1 compared to EAAT2 (Supplementary Table 2). Based on these results we hypothesize that GT951, GTS467, and GTS551 bind and promote glutamate uptake similar to their effects on EAAT2.

### GT951, GTS467, and GTS551 alleviate locomotor deficits in *Drosophila* Htt(128Q) larvae

A robust and widely used model for HD has been generated by expressing full-length human huntingtin gene (htt) with 128 poly-Q repeats (128Q) pan-neuronally using Gal4/UAS targeted gene expression system (Romero et al., 2008). The resulting *Drosophila* Htt(128Q) model exhibits several disease-relevant phenotypes starting at an early larval developmental stage, such as neurodegeneration, locomotor deficits, shortened lifespan and learning and memory impairment (Romero et al., 2008; Beaver et al., 2020).

**FIGURE 1:**
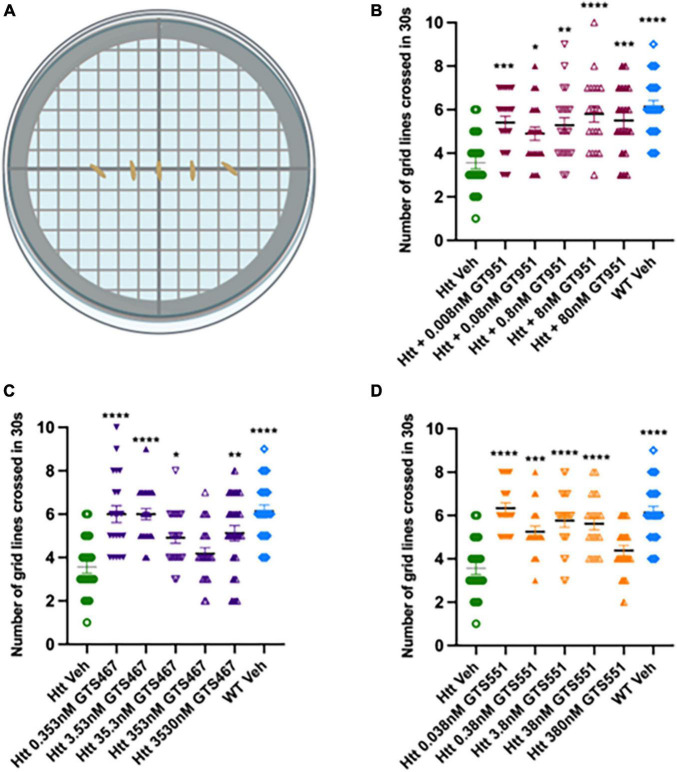
GT951, GTS467, and GTS551 oral administration improves locomotion in *Drosophila* Htt(128Q) larvae. **(A)** Representative schematic of the line crossing assay. Larvae are placed in the center of agar plates for acclimatization and the number of grid lines crossed in 30 s is recorded. **(B–D)** Locomotor results for wild-type larvae fed with vehicle (blue), Htt(128Q) larvae fed with vehicle (green), or dose-dependent drug solutions: GT951 (**B**, maroon), GTS467 (**C**, purple), and GTS551 (**D**, orange). Statistical significance was calculated using one-way ANOVA with Dunnett’s multiple comparisons (*n* > 20). GT951: *F*(6,139) = 6.582, *p* < 0.0001; GTS467: *F*(6,148) = 10.10, *p* < 0.0001; GTS551: *F*(6,140) = 12.91, *p* < 0.0001. *p < 0.05; **p < 0.01, ***p < 0.001, and ****p < 0.0001. Error bars indicate SEM.

Here, we test whether EAAT2 activators can rescue locomotor defects in the *Drosophila* Htt(128Q) model. GT951, GTS467, and GTS551 compounds were tested at five concentrations relative to their EC_50_ in *in vitro* assays (0.8, 35.3, and 3.8 nM, respectively). Larval progeny was reared on food mixed with solutions containing different concentrations of drugs (GT951, GTS467, and GTS551) or vehicle (DMSO). Larval line crossing assay was performed, and the number of gridlines crossed in 30 s were quantified for wildtype vehicle, HD vehicle, and HD drug treatments (Figure 1A). In agreement with previous studies (Beaver et al., 2020), larvae expressing Htt(128Q) showed significantly reduced locomotion as compared to wild-type larvae. GT951 administration successfully rescued locomotor function in Htt(128Q) larvae at all five concentrations with 8 nM (one-fold over EC_50_) being the most effective concentration closest to wild type phenotype (Figure 1B). Similarly, significant increase in locomotor ability was observed with most GTS467 and GTS551 concentrations with 3.53 nM (one-fold below EC_50_) and 0.038 nM (two-folds below EC_50_) being the most effective concentration, respectively (Figures 1C, D). These results suggest that GTS467 and GTS551 have better performance than GT951 *in vivo*. Additionally, there is no indication of adverse toxicity even at high concentrations, suggesting the drugs are safe *in vivo*. Overall, all three compounds can rescue locomotor behavior in *Drosophila* Htt(128Q) larvae over a wide range of doses.

### GT951, GTS467, and GTS551 improve associative learning and memory retention in *Drosophila* Htt(128Q) larvae

In *Drosophila*, olfactory learning and memory is well characterized and predominantly mediated by N-methyl-D-aspartate (NMDA) glutamate receptors in the mushroom body of central nervous system (Margulies et al., 2005; Xia et al., 2005). In accordance with glutamate excitotoxicity-associated cognitive deficits in HD (Estrada Sánchez et al., 2008), *Drosophila* Htt(128Q) larvae exhibit significant olfactory associative learning and STM loss (Beaver et al., 2020). To test for EAAT2 activation-mediated glutamate clearance and cognitive rescue, we utilized the larval olfactory learning and memory assay. Based on the locomotor assay readout, the most effective *in vivo* drug concentrations were selected for each drug (8 nM for GT951, 3.53 nM for GTS467, and 0.038 nM for GTS551). As sensory reflexes tend to be heavily implicated in the learning and memory function, we first evaluated for intact-olfactory and gustatory reflexes across all treatments (Supplementary Figure 1). Although speed of GTS467-treated larvae were significantly increased, final ΔRI values for all genotypes have been normalized with their respective speed.

**FIGURE 2:**
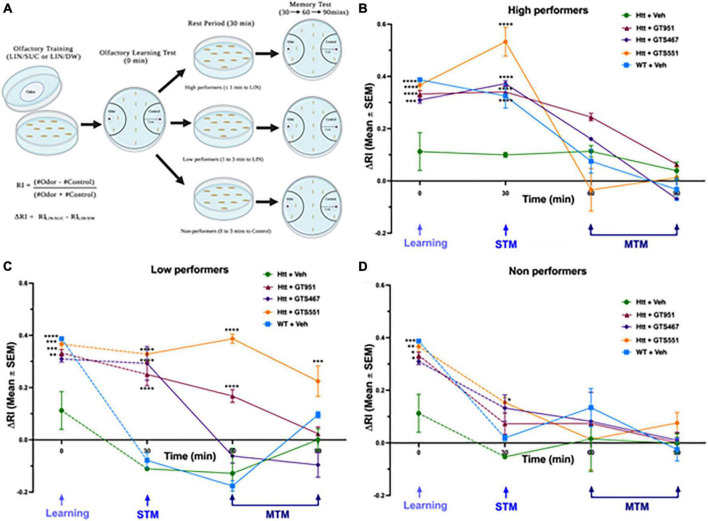
GT951, GTS467, and GTS551 rescue associative learning in all groups and memory retention in high- and low-performers. **(A)** Representative schematic of the improved olfactory learning and memory assay. Third instar larvae underwent olfactory training for 30 min on agar plates with odorant linalool and either sucrose (LIN/SUC) or distilled water (LIN/DW). Olfactory learning test (0 min) was conducted, and larvae were separated into high-, low-, and non-performers for testing STM (30 min) and MTM (60 and 90 min). Response index (RI) was calculated as (number of larvae in the odor area–number of larvae in the control area)/total number for larvae counted. Delta RI (ΔRI) was calculated as the difference between RI(LIN/SUC) and RI(LIN/DW) with speed normalization for **(B)** high-, **(C)** low-, and **(D)** non-performers. Statistical significance was calculated using two-way ANOVA with Dunnett’s multiple comparison test (*n* = 40–50 larvae per run, 8 runs per condition). *p < 0.05; **p < 0.01, ***p < 0.001, and ****p < 0.0001. For high performers, an overall treatment [*F*(4,145) = 10.13, *p* < 0.0001], time [*F*(3,145) = 91.09, *p* < 0.0001], and interaction [*F*(12,145) = 9.029, *p* < 0.0001] effect was detected. For low-performers, an overall treatment [*F*(4,192) = 44.15, *p* < 0.0001], time [*F*(3,192) = 49.79, *p* < 0.0001], and interaction [*F*(12,192) = 13.18, *p* < 0.0001] effect was detected. For non-performers, an overall treatment [*F*(4,151) = 3.477, *p* = 0.0095] and time [*F*(3,151) = 32.34, *p* < 0.0001] effect was detected, while no overall interaction effect was detected [*F*(12,151) = 1.394, *p* = 0.1745]. Error bars indicate SEM.

In our experimental setup, larvae are first trained to associate linalool odor (LIN) with a gustatory reinforcer, sucrose (SUC) and then their olfactory response is tested for learning (0-min), short-term memory (STM, 30 min), or middle-term memory (MTM, 60 and 90 min) (Margulies et al., 2005). To account for any natural preferences to the odor, larvae are also separately trained on a control setup containing linalool with distilled water (LIN/DW). The final delta response index (ΔRI) is calculated as the difference between responses in LIN/SUC and LIN/DW (Figure 2A; Honjo and Furukubo-Tokunaga, 2005; Beaver et al., 2020). In olfactory learning test at 0 min, vehicle-treated wild type larvae had a positive ΔRI of 0.39, suggesting the larvae strongly associated the linalool odor with sucrose (Figures 2B–D). In contrast, vehicle-treated Htt(128Q) larvae exhibited significant loss in learning with ΔRI of 0.11, suggesting the larvae only had a mild association of odor with sucrose. Strikingly, treatment with all three drugs, GT951, GTS467, and GTS551, significantly improved learning in the Htt(128Q) larvae with ΔRI of 0.33, 0.31, and 0.36, respectively (Figures 2B–D), suggesting these drugs have the potential to restore learning defects associated with HD.

After the olfactory learning test, larvae from each genotype were divided into three sub-groups based on their performance: high-performers (odor side, ≤1 min), low-performers (odor side, 1–3 min), and non-performers (non-odor side, 0–3 min) (Figure 2A). Similar to the learning test, memory retention was evaluated in terms of delta response index (ΔRI). In high-performers, Htt(128Q) larvae exhibited significantly less STM at 30 min (ΔRI 0.1) as compared to the wild-type larvae (ΔRI 0.36) (Figure 2B). Treatment with all three compounds completely rescued STM at 30 min, with GTS551 showing the most prominent rescue (ΔRI 0.52) (Figure 2B). The MTM of all treatment groups were comparable without any significant deviations. In low-performers, although both wild-type and Htt(128Q) larvae exhibited low STM at 30 min (ΔRI −0.08 and −0.11, respectively), only the wild-type larvae showed a trend for positive association in the experimental LIN/SUC setup (Supplementary Figure 2). Similar to the high performers, treatment with all three drugs alleviated STM in low performers at 30 min (ΔRI 0.25 to 0.33), with some drugs showing prolonged rescue effects (Figure 2C). Both, GT951, and GTS551, improved MTM of low-performing Htt(128Q) larvae at 60 min while GTS551 was effective even at 90 min. Contrary to the high- and low-performers, the drugs did not impact STM or MTM in non-performers except for GTS551 improved STM in Htt(128Q) larvae at 30 min (Figure 2D). In conclusion, these results underscore the effectiveness of GT951, GTS467, and GTS551 in restoring associative learning and STM in high- and low-performers, while GT951 and GTS551 alleviate MTM in low-performers as well. Therefore, these novel EAAT2 activators provide an exciting new strategy for treatment of cognitive function in the early stages of HD pathogenesis.

### GT951 and GTS551 protect against early mortality in *Drosophila* adult flies expressing Htt(128Q)

**FIGURE 3:**
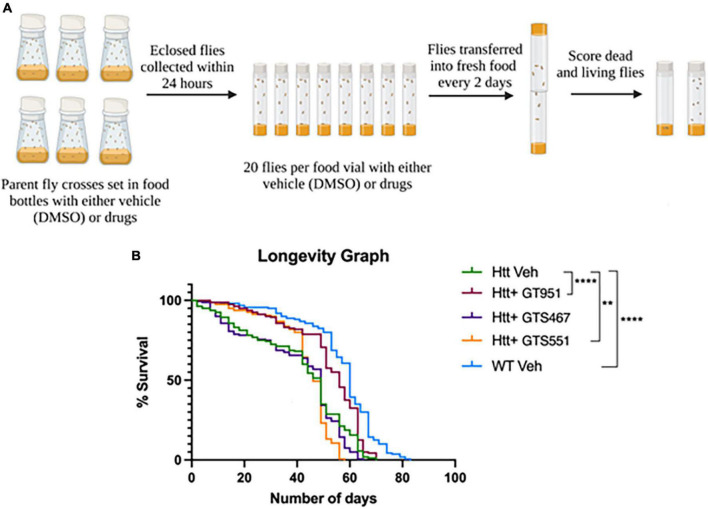
Effect of GT951, GTS467, and GTS551 on survival of *Drosophila* Htt(128Q) adult flies. **(A)** Representative schematic of the longevity assay protocol where all treatment groups were monitored and constantly flipped into fresh food for several weeks. The number of surviving flies in the respective treatment group were recorded during each transfer until no living flies were present. **(B)** Longevity graph depicting the survival percentage of the five treatment groups over a period of 83 days. Statistical significance was calculated using the Log-Rank Mantel-Cox analysis (Chi square = 215.2, dF = 4, *p* < 0.0001, *n* = 160). **p < 0.01 and ****p < 0.0001.

Since all three compound significantly improved neurodegenerative phenotypes in early stages of HD progression, we next assessed their effectiveness over long-term adult survival phenotype. The survival rate of flies expressing Htt(128Q) has been shown to be significantly lower than the controls (Romero et al., 2008), therefore resembling reduced life expectancy in HD patients (Rodrigues et al., 2017). The flies tested in longevity assay were fed vehicle or drugs from early adult stages and were flipped into fresh vials every 2 days until no living flies were present (Figure 3A). The most effective drug concentration for GT951, GTS467, and GTS551 (8, 3.53, and 0.038 nM, respectively) was tested. The survival graph shows a significant reduction in longevity of the adult flies expressing Htt(128Q) as compared to wild type (Figure 3B). While the wild type flies were alive until 83 days with a median survival of 60 days, the Htt(128Q) flies only survived up to 70 days with a median survival of 49 days. Although the total lifespan of Htt(128Q) flies remained unaffected, some drug treatments elevated the survival rates about until mid-life. For example, GT951 drug administration significantly improved median survival to 56 days and was effective until Day 63 on which 20% flies perished in a single day (*p* < 0.0001). Similarly, GTS551 treatment significantly improved survival rates of the population up until Day 42 when 15.6% of flies perished in a single day (*p* < 0.01). Unlike other two compounds, GTS467 did not have an impact on the longevity or survival rates of the Htt(128Q) flies. Therefore, these findings reveal that at their most effective concentrations, GT951 and GTS551 partially protect against early mortality in *Drosophila* Htt(128Q) adult flies.

### GT951, GTS467, and GTS551 upregulate neural expression of EAAT2 homolog and cognition-associated genes in *Drosophila* Htt(128Q) larvae

**FIGURE 4:**
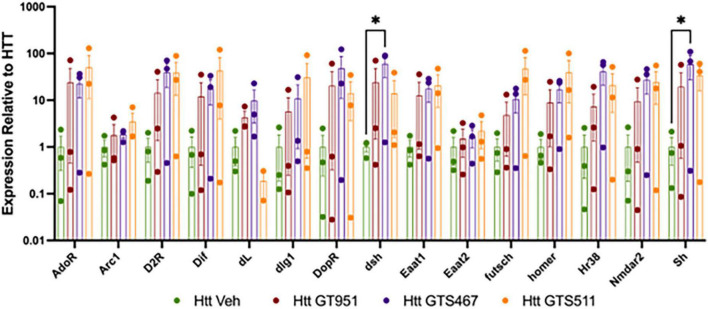
Effect of GT951, GTS467, and GTS551 on Htt(128Q) *Drosophila* larval head gene expression. Expression of learning and memory associated genes from larval heads treated with compounds was assessed via qRT-PCR. Expression was normalized to the vehicle-treated Htt(128Q) larval group. An overall treatment effect was detected via two-way ANOVA [*F*(3,119) = 8.235, *p* < 0.0001]. No overall gene [*F*(14,119) = 1.043, *p* = 0.416] or interaction [*F*(42,119) = 0.5524, *p* = 0.9851] effect was detected. Dunnett’s multiple comparison test was used to compare each treatment to the Htt(128Q) larvae control group for each gene (*n* = 30 brains per replicate, 3 replicates). *p < 0.05. Error bars indicate SEM.

We next assessed if GT951, GTS467, and GTS551 have the ability to epigenetically upregulate expression of glutamate transporters and learning/memory associated genes in our *Drosophila* Htt(128Q) larvae. In *Drosophila*, there are two excitatory amino-acid transporters, the mammalian EAAT2 homolog glutamate transporter dEAAT1, and aspartate/taurine transporter dEAAT2, with dEAAT1 being the only high affinity glutamate transporter in *Drosophila* that is expressed at all developmental stages (Besson et al., 2000; Liévens et al., 2005). Accordingly, *Drosophila EAAT1* knockdown has been shown to induce glutamate-mediated excitotoxicity, locomotor deficits and reduced longevity (Rival et al., 2004; Peng et al., 2019). Similarly, we included *Drosophila* homologs of major mammalian learning and memory genes that have been shown to affect cognitive behavior in *Drosophila* (Croning et al., 2009; Beaver et al., 2020). Larval progeny expressing Htt(128Q) in the brain were reared on food containing either the most effective drug concentration or DMSO vehicle and the heads were dissected for neural gene expression analysis (Figure 4). We find an overall effect of treatment with compounds in the 15 gene panel via two-way ANOVA (*F* = 8.235, *p* < 0.0001). Compared to vehicle-treated larvae, compound treatments resulted in trending increases in gene expression for most comparisons. We find that GTS467 significantly induced expression of in the Wnt signaling pathway gene disheveled (*dsh*) (*p* < 0.05) and the potassium ion channel gene shaker (*sh*) (*p* < 0.05). Additionally, treatment with all three compounds resulted in a trending increase in the *Drosophila EAAT1* (mammalian EAAT2 homolog), suggesting these compounds may be able to upregulate glutamate transporters and genes relevant to cognition under disease conditions.

## Discussion

Huntington’s disease is associated with neurodegeneration of the striatum, an area of the brain that affects locomotor and cognitive functions (Aylward et al., 2011). Mutant Htt protein aggregates directly affect the MSN of the striatum (Saudou and Humbert, 2016). Although multiple studies over the past decade have made compelling arguments for the causal role of glutamate excitotoxicity in the neurodegeneration of the MSNs in the striatum (Sepers and Raymond, 2014), current anti-excitotoxic drugs, such as memantine, improved motor symptoms but did not improve measures of cognition (Karki et al., 2014a). In normal termination of glutamate neurotransmission, glutamate is removed from the synapse by EAAT2 a glutamate transporter predominantly present on astrocytes. In several neurodegenerative disease states including HD, EAAT2 is downregulated which results in accumulation of glutamate causing excitotoxicity characterized by excessive activation of postsynaptic glutamate receptors which eventually leads to apoptotic or necrotic cell death (Lau and Tymianski, 2010). Previous studies have shown that the EAAT2 is downregulated in an HD transgenic mouse model and activating the expression of EAAT2 shows rescuing of neurodegenerative phenotypes in an HD mouse model (Behrens et al., 2002; Bradford et al., 2009) implicating its neuroprotective role in glutamate uptake (Pajarillo et al., 2019). Therefore, activation of EAAT2 presents an exciting new therapeutic strategy for preventing neuronal excitotoxicity mediated neurodegeneration that remains to be fully explored.

Here, we tested three EAAT2 activators, namely, GT951, GTS467, and GTS551 for the first time *in vivo* in the well characterized *Drosophila* HD model expressing 128 poly-Q repeats (128Q) pan-neuronally (Romero et al., 2008; Beaver et al., 2020). GT951 stimulates the transport of glutamate by interacting with residues that form the interface between the trimerization and the transport domains. In order to increase the likelihood of the drug passing through the blood-brain barrier, we optimized the drug-like properties of GT951 and designed additional compounds GTS467 and GTS551 with better drug-like and pharmacokinetic profiles (Das et al., 2022).

In HD, there is a preferential neurodegeneration of the MSNs in the striatum, which is considered a control center for motor function that receives convergent inputs from the cerebral cortex, thalamus, and hippocampus for voluntary movement and dysregulation of neurotransmission at these MSNs lead to chorea like symptoms (Dunnett et al., 2005). Because progression of HD pathogenesis is commonly characterized by escalation of motor deficits in humans (Dorsey et al., 2013; Plotkin and Surmeier, 2015), we first assessed the effect of GT951, GTS467, and GTS551 in rescuing locomotor deficits observed in the early stages of our *Drosophila* HD model. Interestingly, all three compounds significantly improved locomotor function of HD larvae at five different ten-fold diluted nanomolar concentrations, suggesting that EAAT2 activators have the potential to rescuing locomotor behavior *in vivo* over a wide range of doses.

In addition to motor deficits, there are also cognitive deficits that are commonly seen in HD. Two important receptors for learning and memory are the AMPA and the NMDA receptors (Warburton et al., 2013; Diering and Huganir, 2018). These receptors play a role in forming memories through short term potentiation and long term potentiation (Warburton et al., 2013; Diering and Huganir, 2018). While these receptors allow for the fast transmission of glutamate, they can be overstimulated under conditions of excitotoxicity leading to among others cognitive impairment (Dunnett et al., 2005; Warburton et al., 2013; Diering and Huganir, 2018). With these cognitive deficits in learning and memory in mind, we assessed the ability of GT951, GTS467, and GTS551 to rescue associative learning and memory in early stages of HD progression in our *Drosophila* HD model. Using the most effective drug concentrations obtained from the locomotion assay for each drug, we used an olfactory learning and memory assay that tested for associative learning, STM and MTM. In the Htt(128Q) larvae with vehicle treatment, we observed a deficit in learning as compared to our wildtype control, therefore recapitulating cognitive decline in HD patients. Treatment with all three drugs: GT951, GTS467, or GTS551 showed significant improvements in larval associative learning, suggesting these drugs have the potential to restore learning deficits associated with HD. After the initial learning phase of the assay, we tested to see how well memory was sustained at the 30 min (STM), 60 min (MTM), and 90 min (MTM) timepoints. Based on the learning phase, the larvae were split into three groups, high performers (those that traversed to the odor side ≤1 min), low-performers (traversed to odor side in 1–3 min) and non-performers (those that remained at the non-odor side during the 0–3 min). In the HD model, we observed significantly lower STM in the high performers when compared to the control. In high and low performers treated with all three drugs, we observed a rescue of STM. In low performers, GT951 and GTS551 improved MTM at 60 min and GTS551 even showed rescue at 90 min. In contrast with the high and low performers, the drug did not affect the STM or MTM of non-performers, other than improved STM at 30 min for the HD model treated with GTS551. Overall, the learning and memory assays show that treatment with all three drugs improved learning and STM for high and low performers, and treatment with GT951 and GTS551 improved MTM in low performers as well. It is likely that these EAAT2 activators reduce excitotoxicity and promote normal learning and memory behaviors. More studies are required to understand the molecular mechanisms underlying the effects of these compounds on HD pathogenesis.

Huntington’s disease is also associated with a significant decrease in life expectancy and is usually lethal within 10 to 30 years after the onset of symptoms (Dorsey et al., 2013). To see how these drugs might improve survival rate, we assessed the longevity of our *Drosophila* Htt(128Q) adult flies treated with the three compounds. The HD vehicle-treated flies showed reduced longevity when compared to the control flies. Treatment with GT951 significantly improved the median survival of the HD flies from 49 to 56 days while treatment with GTS551 improved survival rates until Day 42. These results underscore the efficacy of these drugs in improving HD-associated longevity defects and support their use in treatment of HD.

Dysregulation of glutamate uptake by EAAT2 in several neurodegenerative diseases including HD has been attributed to inactivity of EAAT2 which could be due to reduced transcription of *EAAT2*, reduced expression of EAAT2 on the membrane, or increased degradation of EAAT2 (Hirschberg et al., 2021). Further, EAAT2 activators such as ceftriaxone are known to improve glutamate uptake activity by increasing EAAT2 protein expression on the membrane (Lee et al., 2008). Previous studies have found that HDACs work as co-repressors with a transcription factor called Ying Yang 1 (YY1) to repress the expression of EAAT2 in astrocytes (Karki et al., 2014a). When the NF-kB pathway is activated, it binds to the YY1 promoter which upregulates YY1 expression. The upregulated YY1 then uses HDACs as co-repressors to reduce expression of EAAT2. Additionally, YY1 interacts with astrocytic NF-kB and inhibits its positive regulatory function of EAAT2 (Karki et al., 2014a). We have previously shown our EAAT2 activators, exemplified by GT951, reduce glutamate excitotoxicity through positive allosteric modulation of the EAAT2 receptor which increases the rate of glutamate removal from the synapse (Kortagere et al., 2017). In our studies we found expression of *dEAAT1*, which is the *Drosophila* homologue of mammalian *EAAT2*, showed trending improvement with all three compound treatments when compared with vehicle treatment similar to what has been reported in a mouse model of HD (Behrens et al., 2002; Bradford et al., 2009). However, the additional gene listed as *dEAAT2* was found to have no change in expression under these compound treatments which needs to be further validated. These results suggest that EAAT2 activation may involve one or more of the mechanisms highlighted previously and these allosteric modulators in addition to stabilizing the substrate binding conformation of EAAT2 may also promote increased expression on the membrane. In addition to *dEAAT1*, we found elevated expression of the Wnt signaling pathway gene *dsh* and the potassium ion channel gene *sh* (Figure 4). Our previous studies have demonstrated that these genes are involved in promoting neuroplasticity, learning, and memory regulation in *Drosophila* and are epigenetically regulated by Tip60/HDAC2 (Panikker et al., 2018). These genes are implicated in neuronal development and differentiation, hyperkinetic movements, mushroom body dependent activities such as sleep, learning and memory regulation in *Drosophila* (Packard et al., 2002; Gasque et al., 2005; Bushey et al., 2007; Miech et al., 2008). The mammalian orthologs of these genes, especially DVL2 and KCNA1/2, are implicated in epilepsy and episodic ataxia which are associated with both glutamate excitotoxicity and hippocampal based cognitive deficits (Aloi et al., 2022; Samanta, 2022; Muller et al., 2023). While results from our studies provide correlational evidence for the likely association of these genes with glutamate regulation by EAAT2 activators, further studies are needed to confirm these associations and understand any causal implications in neurodegenerative diseases.

Our results show that GT951, GTS467, and GTS551 show promise in reducing glutamate excitotoxicity associated phenotypes through epigenetic regulation of EAAT2 gene expression in the *Drosophila* HD model. There are multiple clinical trials that are currently aimed at treating the symptoms associated with HD (Ondo et al., 2007; Kim et al., 2021) and glutamate excitotoxicity may be one of the causal factors for the pathogenesis and neurodegeneration in HD. Additionally, this study also demonstrated that the novel EAAT2 activators can treat both motor and cognitive symptoms while also preserving longevity. Future work in a mammalian species will be warranted to demonstrate efficacy and safety for further development of these compounds as therapeutics for HD.

## Data availability statement

The original contributions presented in this study are included in the article/Supplementary material, further inquiries can be directed to the corresponding author.

## Ethics statement

Ethical review and approval was not required for the study of animals in accordance with the local legislation and institutional requirements.

## Author contributions

SK developed the hypothesis and study design. AB, VP, NB, and FB developed the protocols and performed all the experiments and data analysis, SK and FE helped with troubleshooting the experiments. All authors contributed to writing, formatting, and editing the manuscript.
