# Supplementary material for: Novel EAAT2 activators improve motor and cognitive impairment in a transgenic model of Huntington’s disease
Source: Front Behav Neurosci. 2023 Jun 7;17:1176777. doi: 10.3389/fnbeh.2023.1176777 (PMC10282606; doi:10.3389/fnbeh.2023.1176777)
Supplement: Supplementary file 1 [file Data_Sheet_1.docx]

**
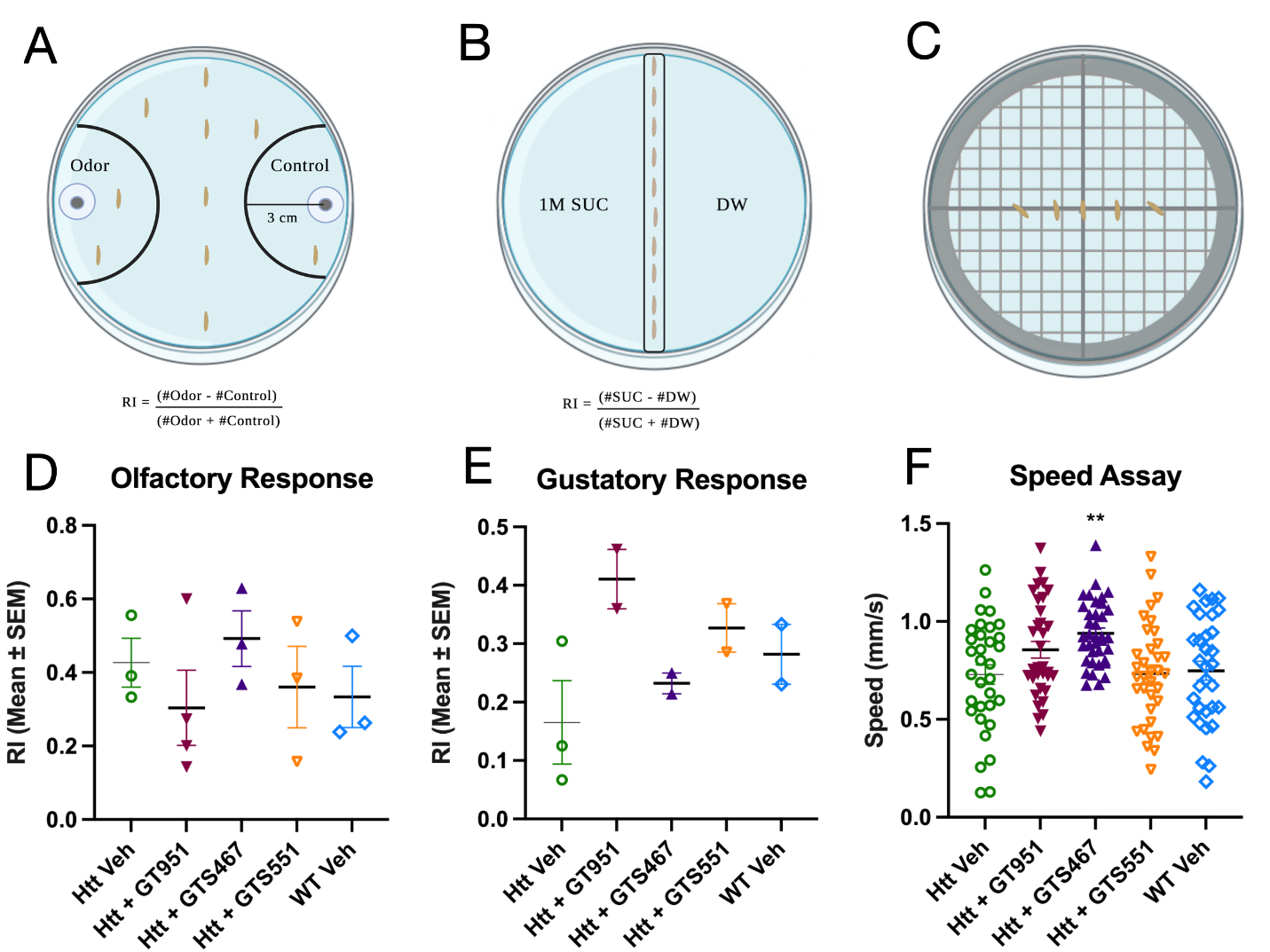
Supplementary Figure 1. Larval sensory reflexes remain mostly intact post GT951, GTS467 and GTS551 administration.** Representative schematics of (A) olfactory, (B) gustatory, and (C) speed assays, which were conducted to evaluate for intact sensory reflexes post drug or vehicle administration. Response index (RI) was calculated for olfactory and gustatory assays by calculating the number of larvae that moved to the desired odor or sucrose side, respectively, over the total number of larvae tested. For speed analysis, Tracker software was used to analyze the larval locomotor speed in mm/s that will be utilized to normalize for ΔRI values in the subsequent learning and memory experiments. Statistical significance was calculated one-way ANOVA with Dunnett’s multiple comparisons. ** p<0.01. Error bars indicate SEM.

**
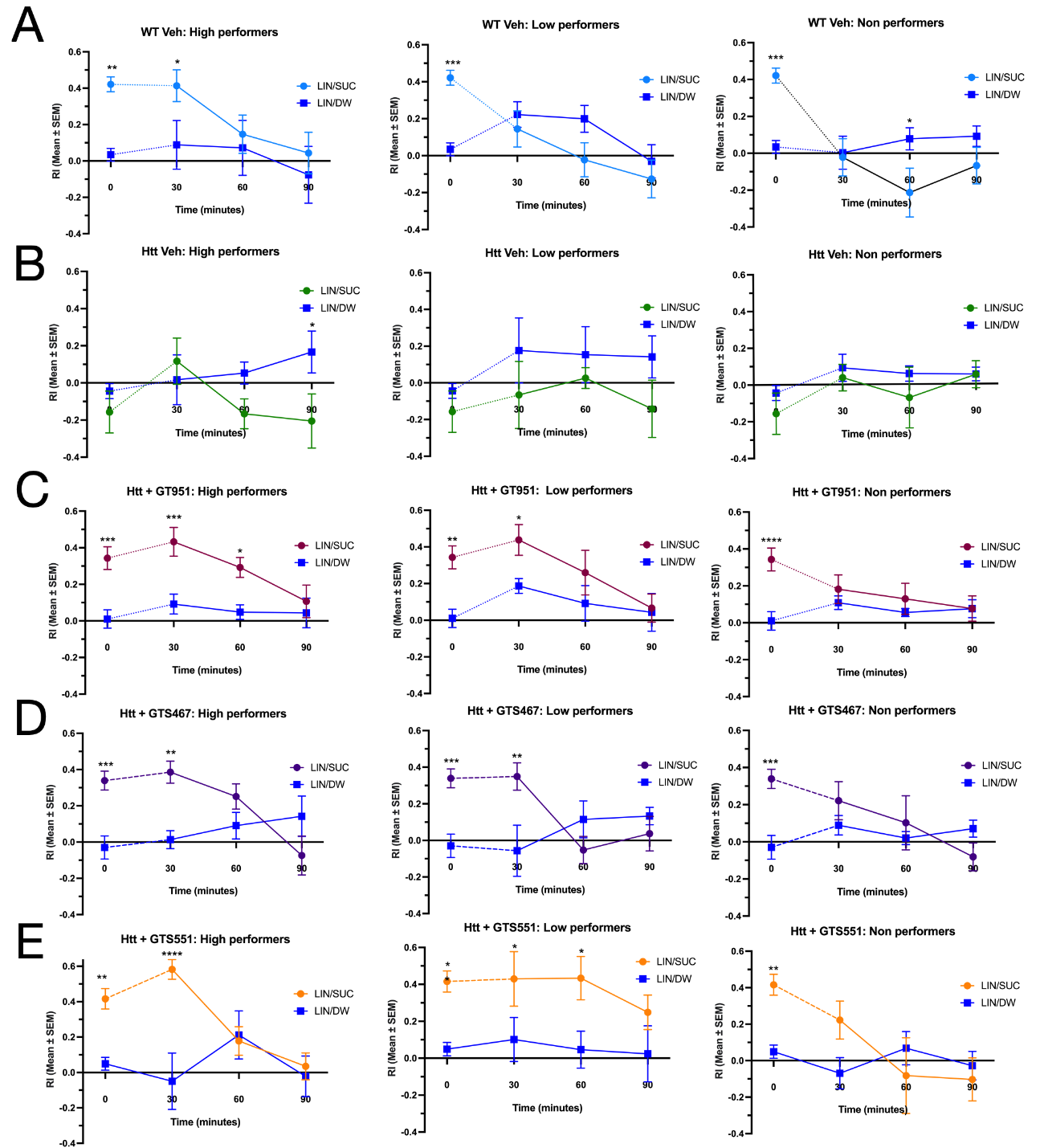
**

**Supplementary Figure 2. Response index (RI) of experimental and control setups in olfactory learning and memory assays.** For each treatment, larvae were either trained on an experimental setup with linalool odor and sucrose (LIN/SUC, black lines) or a control setup with linalool odor and distilled water (LIN/DW, blue lines). The response indexes were calculated for **(A)** wild type with vehicle, **(B)** Htt(128Q) with vehicle, **(C)** Htt(128Q) with GT951, **(D)** Htt(128Q) with GTS467, and **(E)** Htt(128Q) with GTS551.

**Supplementary Table 1: Primer sequences and mammalian homologs for *Drosophila* learning and memory genes tested via qRT-PCR.**

| **Compound** | **dEAAT1** | **EAAT2** |
| --- | --- | --- |
| GT951 | 66.70 | 54.65 |
| GTS467 | 56.20 | 53.50 |
| GTS551 | 58.97 | 51.49 |

**Supplementary Table 2:** Docking scores for GT951, GTS467 and GTS551 docked to dEAAT1 or EAAT2 is shown. All compounds show slightly higher docking scores when bound to dEAAT1 suggesting better binding to dEAAT1.

| **No.** | ***Drosophila* gene** | **Mammalian homolog** | **Forward primer** | **Reverse primer** |
| --- | --- | --- | --- | --- |
| 1. | AdoR | ADORA2A | TCCGAGCTGAACATACCCTAC | GCGTCGCAACTTTCTTTCCC |
| 2. | Arc1 | ARC | ATGGCCCAGCTTACACAGATG | GGAGAAGTTGCCTTTGCCTC |
| 3. | Dif | RELB | AGCACAACACCATAGGCTCC | CGACCGCTGGATAGGAAGAC |
| 4. | D2R | DRD2 | CCGCGTTTGCCTTACGATACT | TGTTGTTGAGGCCCAGAACTAT |
| 5. | dL | REL/RELA | CTGCCATCCTGGTGGTCATT | TGAAACCGAAAACCCGATCA |
| 6. | dlg1 | DLG1 | TATTTGCCACCGAACAGGCGTTG | ACCCAAATGGTTGGTCCCGACT |
| 7. | DopR | DRD5 | GCATCTTCCTATCGGTGCTGA | AGATCCGCAATCGCTAACGAG |
| 8. | dsh | DVL3 | ACCCAGCCGCAACGAGTCAA | AACCGATTGCTGCCGGACAC |
| 9. | Eaat1 | SLC1A3 | CGAGTCAGGTTCTACTGCCG | CCTAATCGAAACGGGGGAGG |
| 10. | Eaat2 | SLC1A2 | AGGTCGCGGTCTTAACTGC | CTGTTCAATTGGCGAGCCC |
| 11. | futsch | MAP1A/B | CCGCGGCTGAGCAATCCGCCC | GGCGCTTCAGTTTTCCCGGCGCA |
| 12. | homer | HOMER2 | GAACAACCGATTTTCACCTGC | GAGCTGTCGTAGAAGAAGCTAAC |
| 13. | Hr38 | NR4A2 | CGGCCACTTCAATGCCATC | TGCGGAAACACCATTATATTCGT |
| 14. | Nmdar2 | GRIN2D | GGCATCCCGGTTATCTCGTG | AGAACTGGTGCCACTTGTAGC |
| 15. | Sh | KCNA1 | GGTGGCATGGCCGCCGTTGC | CTCGAGCTGCTCCTTCTGGTG |
| 16. | RpL32 | RPL32 | AAACGCGGTTCTGCATGAG | GACGCTTCAAGGGACAGTATCTG |
